# Assessment of Mannitol-Induced Chronic Blood–Brain Barrier Dysfunction In Vivo Using Magnetic Resonance

**DOI:** 10.3390/ijms25189792

**Published:** 2024-09-10

**Authors:** Ana Sampedro-Viana, Sabela Fernández-Rodicio, José Castillo, Pablo Hervella, María Luz Alonso-Alonso, Ramón Iglesias-Rey

**Affiliations:** Neuroimaging and Biotechnology Laboratory (NOBEL), Clinical Neurosciences Research Laboratory (LINC), Health Research Institute of Santiago de Compostela (IDIS), Hospital Clínico Universitario, Rúa Travesa da Choupana s/n, 15706 Santiago de Compostela, Spain; ana.sampedro@rai.usc.es (A.S.-V.); sfernandezrodicio@gmail.com (S.F.-R.); jose.castillo.sanchez@sergas.es (J.C.); pablo.hervella.lorenzo@sergas.es (P.H.)

**Keywords:** blood–brain barrier (BBB), brain disorders, gadolinium leakage, mannitol, magnetic resonance imaging (MRI)

## Abstract

The blood–brain barrier (BBB) is essential for protection and plays a crucial role in chronic neurological disorders like small-vessel disease and Alzheimer’s disease. Its complexity poses significant challenges for effective diagnostics and treatments, highlighting the need for novel animal models and comprehensive BBB dysfunction studies. This study investigates chronic BBB dysfunction induction using osmotic disruption via mannitol in healthy adult male Sprague Dawley rats over 12 weeks. Group 1 received 1 bolus/week (2.0 g/kg), Group 2 received 3 boluses/week (1.5 g/kg), and Group 3 received 3 boluses/week (2.5 g/kg). BBB dysfunction was assessed using gadolinium (Gd) infusion and MRI to evaluate location, severity, evolution, and persistence. MR spectroscopy (MRS) examined the brain metabolism changes due to intravenous mannitol, with T2-weighted MRI assessing brain lesions. Biomarkers of neuroinflammation were analyzed in the highest mannitol dose group. Our data show chronic BBB dysfunction primarily in the cortex, hippocampus, and striatum, but not in the corpus callosum of rats under periodic mannitol dosing in groups 1 and 2. MRS identified a distinctive metabolite signature, including changes in alanine, choline, and N-acetyl aspartate in the striatum of Group 1. No significant differences were found in the serum levels of all pro- and anti-inflammatory cytokines analyzed in the high-dose Group 3. This study underscores the feasibility and implications of using osmotic disruption to model chronic BBB dysfunction, offering insights for future neuroprotection and therapeutic strategies research.

## 1. Introduction

The brain endothelium serves as both a biological and mechanical barrier between the brain and vascular compartments and is crucial in linking risk factors to neurological diseases such as lacunar stroke, Alzheimer’s disease, and multiple sclerosis. The vascular endothelium plays an essential role in regulating vascular homeostasis through mechanisms like vasodilation, the inhibition of platelet adhesion and aggregation, anticoagulant and profibrinolytic effects, anti-inflammatory actions, and the inhibition of leukocyte adhesion and migration. In the brain, these mechanisms are mediated by proteins associated with endothelial cells, whose expression or inhibition varies with age and functional state. The integrity or permeability of the blood–brain barrier (BBB), reflecting the normal function of the brain endothelium, can be indirectly assessed through imaging methods and blood biomarkers that indicate cerebral endothelium damage and potential neurological disease. Maintaining BBB integrity is vital for pathophysiological studies, early diagnosis, and the development of preventive treatments for stroke and vascular cognitive impairment [1,2,3].

There are different in vivo models that mimic the processes associated with the origin and progression of BBB dysfunction [4,5]. However, although animal models of chronic BBB dysfunction are invaluable for research, they are limited and have significant disadvantages. Genetic models, such as mice with mutations in amyloid precursor protein (APP), presenilin-1 (PS1), or PDGF-B genes, replicate Alzheimer’s disease pathology and pericyte deficiency, leading to sustained BBB disruption [6,7]. Chemical induction models, including Streptozotocin-induced diabetes and lipopolysaccharide (LPS) exposure, mimic conditions like diabetic encephalopathy and chronic inflammation, respectively [8,9]. Models involving kainic acid, pilocarpine, or experimental autoimmune/allergic encephalomyelitis (EAE) induce seizures or autoimmune responses against myelin, resulting in chronic BBB dysfunction akin to epilepsy or multiple sclerosis [10,11]. Mechanical models like controlled cortical impact (CCI) or middle cerebral artery occlusion simulate traumatic brain injury (TBI) or ischemic stroke, respectively, causing long-term BBB dysfunction [12,13]. Viral models using HIV-1 transgenic rats replicate HIV-associated neurocognitive disorders, while aging models in naturally aged rodents study age-related BBB changes and neurodegenerative conditions [14].

These models collectively provide valuable insights into BBB dysfunction’s pathophysiology for the following: (i) understanding the location, progression, and long-term effects of BBB impairment; (ii) studying comorbidities, as chronic BBB dysfunction often occurs alongside other pathologies such as inflammation, neurodegeneration, and metabolic disorders; (iii) exploring new diagnostic strategies, including identifying biomarkers for early diagnosis, monitoring disease progression, and evaluating treatment responses; and (iv) developing treatments for related neurological conditions. On the other hand, the main intrinsic limitations of these models involve the use of high doses of neurotoxins, which cause extensive neuronal damage beyond BBB dysfunction, complicating the study of specific BBB-related changes. Methods like systemic injections can affect multiple organs, not just the brain, confounding the interpretation of BBB-specific effects. Moreover, regarding models focusing on the development of specific pathologies such as Alzheimer’s disease or epilepsy, while these models are invaluable for understanding the disease process, they are not suitable for other BBB studies that are not directly related to the disease [1,2,3]. Based on the available reports, new animal models and detailed studies on the status and progression of BBB dysfunction are still needed.

Temporary disruption of the BBB has been widely reported to be a prevalent approach for delivering drugs into the CNS from the circulatory system [15,16,17,18]. The developed BBB disruption methods mainly include osmotic disruption, ultrasound disruption, and magnetic disruption. Osmotic stress models in animals, such as mannitol administration, increase cerebral blood volume and mediate the decrease in junction tightness in the BBB. Consequently, the entry of diverse solutes into the brain is facilitated. On the other hand, systemic intravenous (i.v.) administration of mannitol is widely used clinically to reduce cerebral edema [19,20,21]. However, an intra-arterial (i.a.) injection at a sufficiently high concentration (close to the solubility limit of around 1.4 M) causes brain microvascular endothelial cells to shrink, which is sufficient to induce transient BBB opening.

While a transient BBB opening can be readily achieved with a mannitol bolus, the mechanism of action is not fully understood, and the chronic effects of repetitive infusion on the BBB and brain metabolism remain unclear. The aim of this study is to determine whether the periodic intravenous administration of mannitol over 12 weeks in healthy rats can induce chronic BBB dysfunction. To achieve this, different mannitol dosing regimens were evaluated: 1 bolus/week (2.0 g/kg i.v., 25% solution), 3 boluses/week (1.5 g/kg i.v., 25% solution), and 3 boluses/week (2.5 g/kg i.v., 25% solution). Here, we report a sensitive contrast-enhanced magnetic resonance imaging (MRI)-based method that non-invasively detects BBB dysfunction in vivo. Quantification of BBB permeability was performed and the Gd-leakage was calculated for the whole brain, as well as separately for the cortex, hippocampus, striatum, and corpus callosum. Additionally, we investigate whether these mannitol infusions are associated with alterations in brain metabolism linked to chronic BBB dysfunction using MR spectroscopy. Finally, we studied the association of BBB dysfunction with chronic neuroinflammation, the presence of brain lesions, and increased extravasation of Evans blue (EB) dye.

## 2. Results

### 2.1. General Animal Appearance and Weight Loss

**Figure 1:**
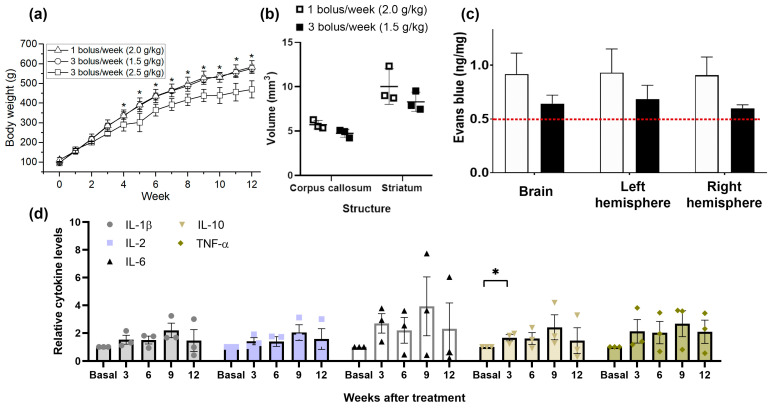
(**a**) Temporal changes in the animals’ weight. (**b**) Corpus callosum and striatum volumes between groups 1 and 3 at 12 weeks. (**c**) Changes in Evans blue extravasation in the entire brain and both hemispheres of rats at the end of the study (*n* = 3 per group). Extravasation of Evans blue was expressed as ng/mg brain tissue. The data are shown as mean ± SEM. The red line indicates normal levels. (**d**) Relative levels of pro-inflammatory cytokines (TNF-α, IL-1β, IL-6, and IL-2) anti-inflammatory cytokines (IL-10) in the mannitol-induced BBB dysfunction. Basal levels represent the measurements taken at week 0, where each analyte value was normalized by dividing it by its corresponding baseline. Values from the subsequent weeks were also normalized to each animal’s baseline. The data are described as the mean ± SEM. * *p* < 0.05.

All animals were visually inspected daily and weighed weekly immediately prior to mannitol administration. Additionally, a pinch test to measure the potential dehydration of the rats was performed daily. No animal showed dehydration signs through the follow-up period. Four weeks after the start of the administrations, a significant weight decrease was observed in Group 3 compared to groups 1 and 2, and this trend continued over time (at 12 weeks: 469.7 ± 43.4 g vs. 575.8 ± 17.4 g vs. 583.8 ± 32.4 g). It should be taken into consideration that high concentration of mannitol could induce lees body fat accumulation, inducing a lower weight gain in Group 3 (3 bolus/week 2.5 g/kg) [22]. However, the animals did not exhibit classical signs of disease behaviors, such as decreased locomotion, stooped posture, and anorexia (Figure 1a).

### 2.2. Presence of Brain Lesions

The results show that repetitive mannitol dosing induces dose-dependent chronic changes in BBB permeability. However, at 12 weeks, no brain lesions or anatomical differences associated with the doses of mannitol administered were found, as evaluated with T2-weighted MRI. Quantitative measurements of the volumes of two studied regions, the corpus callosum and the striatum, revealed no significant differences between the groups (Figure 1b).

### 2.3. BBB Leakage Evaluation

**Figure 2:**
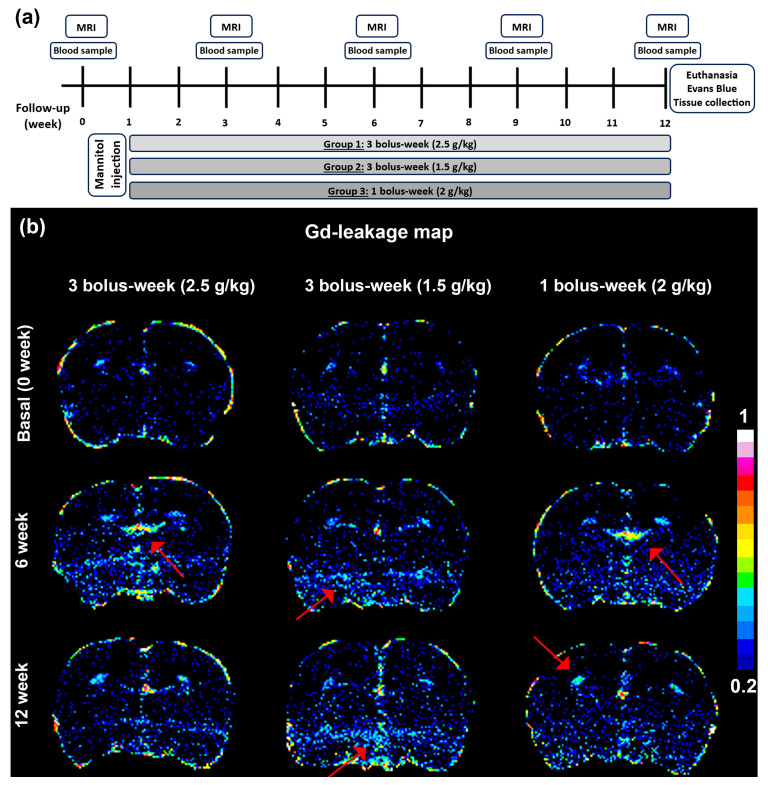
(**a**) Schematic representing the experimental protocol used for measuring the effects of repetitive mannitol i.v. injection on BBB dysfunction. In order to assess BBB permeability, magnetic resonance imaging with Gd contrast enhancement was performed at 0 (basal), 1, 3, and 12 weeks following the beginning of the study. Blood samples were collected at least 24 h after the last bolus at the same time to study inflammatory cytokines. MR spectroscopy was performed at identical time points in both groups 1 and 2, concluding with Evans blue staining. (**b**) Representative axial images of Gd-leakage in the brain at baseline (0 weeks), as well as at 6 and 12 weeks following the beginning of the study. Gd-leakage was calculated as the relative signal enhancement induced by Gd accumulation: the pre-contrast T1-weighted signal intensity was subtracted from the post-contrast signal intensity and divided by the pre-contrast signal intensity, as previously described [23,24]. The greater the number indicated by the color bar in the lower right corner, the closer the color on the image approaches white, indicating greater leakage. No significant Gd enhancement was detected in the brain before mannitol injections, except for the ventricles/circumventricular organs, which have an incomplete BBB. At 6 and 12 weeks, Gd-leakage was still detectable mainly in the hippocampus, striatum, and cortex. Arrows indicate higher extravasation values.

No significant gadolinium (Gd) enhancement was detected in the brain before starting mannitol injections, except for the ventricles/circumventricular organs, which have an incomplete BBB (see Figure 2). A visual analysis revealed that Gd-leakage increased in the weeks following the start of mannitol administrations, mainly in ventral brain regions (superficial gray layer of the superior colliculus, hippocampus, and cortex). We observed varying BBB leakage patterns throughout the study period, with notable leakages occurring predominantly in the cortex, hippocampus, and striatum. At 12 weeks, Gd leakage appears to be less evident in the 3 bolus/week (2.5 g/kg i.v., 25% solution) group compared to the other groups.

**Figure 3:**
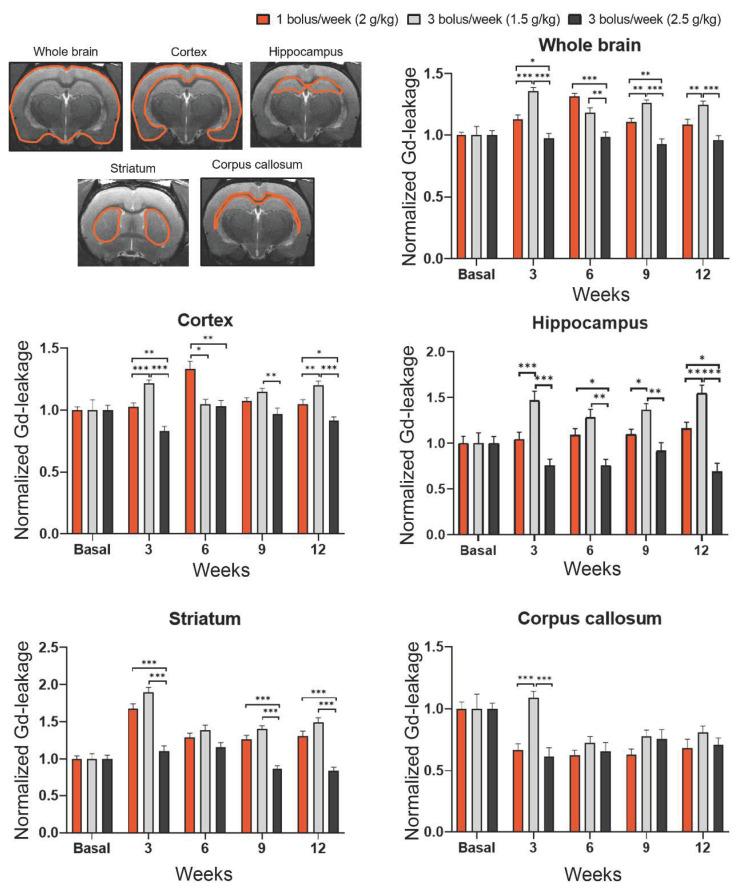
Quantification of Gd-leakage at different follow-up points. T1-weighted MRI was performed to calculate the amount of Gd-leakage per region of interest (ROI) in the brain of the three groups studied. * *p* < 0.05 , ** *p* < 0.01, *** *p* < 0.001 vs. groups, Kruskal–Wallis test followed by Mann–Whitney test.

When assessing the whole brain, Groups 1 and 2 already showed a 10–26% significative increase in Gd leakage at three weeks compared to the values before the injections started. Group 2 exhibited the highest sustained Gd leakage values, with a 26% increase, while Group 3 showed no quantitative increase in Gd extravasation. Indeed, the time course of Gd leakage is sustained over the 12 weeks for Groups 1 and 2, similar to the findings in Group 3, suggesting chronic BBB leakage in the first two groups. Gd leakage became evident in Groups 1 and 2 when analyzing different brain regions separately, with Gd extravasation found in the cortex, hippocampus, and striatum, but not in the corpus callosum. The highest Gd extravasation values were found in the hippocampus and striatum at weeks 3 and 12, showing a 35–60% significant increase at the mannitol dose used in Group 2. Furthermore, when comparing the three groups at each time point, the most significant differences are observed in Group 2 across all evaluated regions and times (Figure 3).

### 2.4. MRI Spectroscopy

To further investigate the BBB chronic dysfunction induced with mannitol dosing, we performed real-time MR spectroscopy (*n* = 3 animals per group). In this study, alanine (Ala), creatine (Cr), g-aminobutyric acid (GABA), glutamate (Glu), glutamine (Gln), glycine (Gly), N-acetylaspartate (NAA), phosphocholine (PCh or Ch), and taurine (Tau) were quantified from two brain regions (cortex and striatum) in each of the two first groups. Metabolites were analyzed for differences between groups, as well as between subjects before and after the mannitol dosing period. Briefly, metabolites were identified (water resonates at 4.7 ppm), and peaks were normalized to the lipid peak areas for each single spectrum. Basal levels represent the measurements taken at week 0 (before treatment), while the mannitol dosing periods correspond to the average metabolite levels observed in the subsequent weeks.

**Figure 4:**
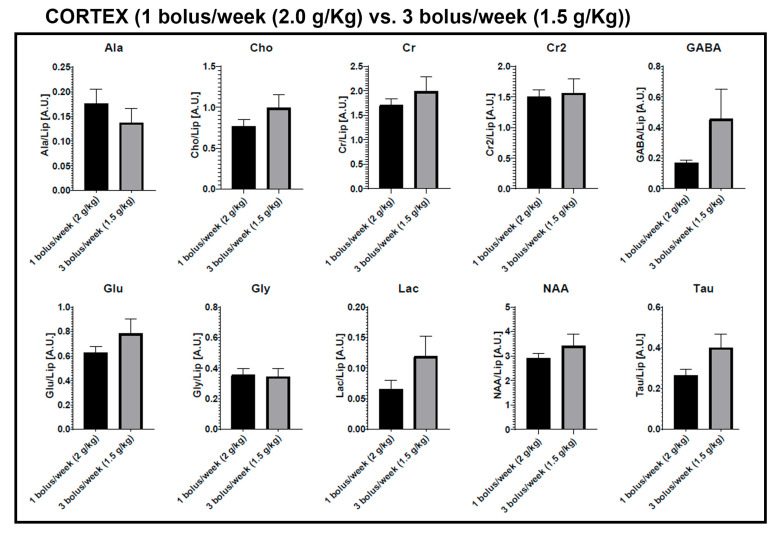

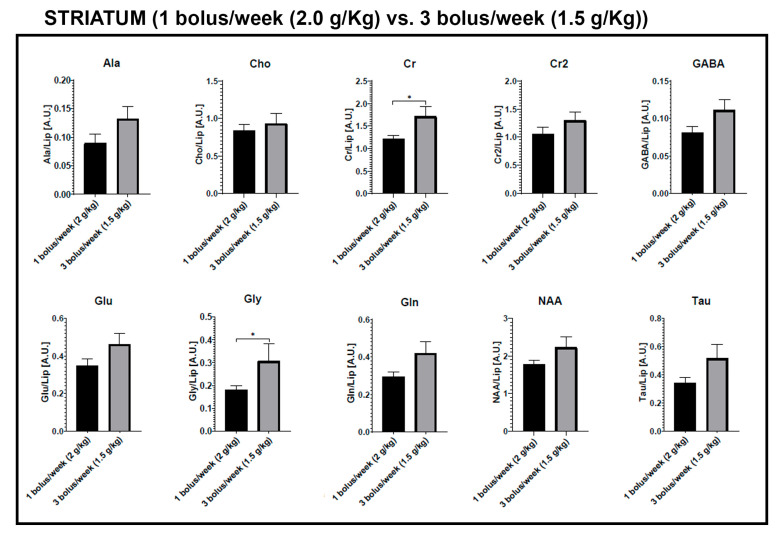
Metabolite differences between Group 1 (1 bolus/week (2.0 g/kg i.v., 25% solution)) and Group 2 (3 bolus/week (1.5 g/kg i.v., 25% solution)), * *p* < 0.05. Metabolites: alanine (Ala), creatine (Cr), g-aminobutyric acid (GABA), glutamate (Glu), glutamine (Gln), glycine (Gly), N-acetylaspartate (NAA), phosphocholine (PCh or Ch), and taurine (Tau). Mannitol dosing periods correspond to the average metabolite levels observed in the subsequent weeks.

**Figure 5:**
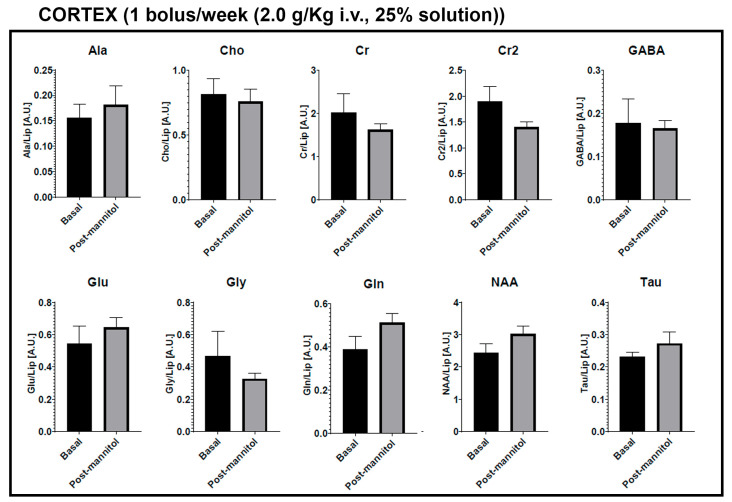

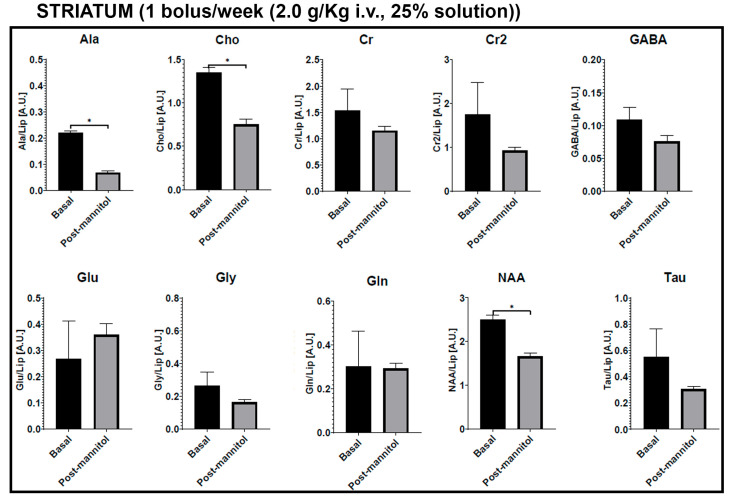
Metabolite differences in Group 1 (1 bolus/week (2.0 g/kg i.v., 25% solution)), * *p* < 0.05. Metabolites: alanine (Ala), creatine (Cr), g-aminobutyric acid (GABA), glutamate (Glu), glutamine (Gln), glycine (Gly), N-acetylaspartate (NAA), phosphocholine (PCh or Ch), and taurine (Tau). Basal levels represent the measurements taken at week 0, while the post-mannitol periods correspond to the average metabolite levels observed in the subsequent weeks.

For analyses between the two groups (1 bolus/week at 2.0 g/kg vs. 3 bolus/week at 1.5 g/kg), there were significant differences for Gly and Cr levels in the striatum. Although no significant differences were found in the cortex, it is notable that the evaluated metabolites show higher values in the 3 bolus per week group (except for Ala and Gly) (Figure 4). Significant relationships were observed between baseline levels and the mannitol dosing periods in the striatum for the metabolites Ala, Cho, and NAA in the group receiving 1 bolus per week at 2.0 g/kg. A decrease of at least 50% compared to the beginning of the study was observed for all three metabolites. On the other hand, no significant differences were found in the cortex (Figure 5).

Finally, there were no metabolite-significant relationships between the basal and mannitol dosing periods in the cortex or striatum in the group of 3 bolus/week at 1.5 g/kg. However, it is noteworthy that except for Gln in the cortex and Cr in the striatum, the metabolites show elevated levels once the study with mannitol injections has started (Figure S1).

### 2.5. Evans Blue

The permeability of the BBB was also evaluated and confirmed by the extravasation of Evans blue dye in each of the two first groups (*n* = 3 per group). Consistent with previous reports, Evans blue (2% *w*/*v* i.v. bolus) rendered the rat’s eyes, ears, nose, and paws dark blue. Changes in Evans blue extravasation in the entire brain and both hemispheres of rats at the end of the study agree with the MRI results and show elevated values compared to healthy rats [25,26]. On average, the increase in extravasation of Evans blue is 58% for Group 1 and 18% for Group 2. Although there are no significant differences between the two groups, the highest values were found in the 1 bolus/week (2.0 g/kg i.v., 25% solution) group (Figure 1c).

### 2.6. Analysis of Pro- and Anti-Inflammatory Cytokines

To investigate the pro-inflammatory reaction in rats subjected to repetitive mannitol administration, we measured the serum levels of pro-inflammatory cytokines such as TNF-α, IL-1β, IL-6, and IL-2 in the high-dose group (3 bolus/week at 2.5 g/kg i.v., 25% solution). Although no significant differences were found, the concentration of all pro-inflammatory cytokines were higher in the mannitol-induced rats than at baseline. As shown in Figure 2d, IL-6 levels increased by 200–400% compared to baseline values. Additionally, all pro-inflammatory cytokines peaked at 9 weeks.

Subsequently, we evaluated IL-10 concentration, which is an important anti-inflammatory cytokine in the mannitol-induced rats. The serum levels of IL-10 in rats undergoing mannitol administration for 12 consecutive weeks showed no significant differences compared to baseline. Although an increase in IL-10 can be observed at week 9, the levels measured throughout the follow-up remained similar to the baseline levels.

## 3. Discussion

In the present study, we investigated the location, severity, evolution, and persistence of BBB dysfunction and associated neuroinflammation following periodic mannitol dosing, as well as its potential contribution to brain damage. BBB leakage was quantified using the step-down infusion of Gd in combination with T1-weighted MRI (post–pre approach) and MR spectroscopy [12,23,24]. This step-down infusion protocol has been used previously in animal models of TBI and epileptogenesis in rats; however, it has never been applied to chronic BBB dysfunction models. It results in a rapid increase in the blood concentration of the contrast agent, which is then maintained at a constant level throughout the infusion.

We found that BBB leakage could be detected and quantified through periodic i.v. administration of mannitol over 12 weeks, confirmed by post-mortem examination using Evans blue. Notably, we observed non-uniform BBB leakage throughout the study period, with the most significant leakage occurring in the cortex, hippocampus, and striatum. The heterogeneous of BBB leakage—occurring in specific brain regions and varying within those regions—could be relevant for future therapies aimed at restoring the BBB. This variability must be considered when evaluating the role of the BBB in the development of different neurological diseases models. The increase in Gd-extravasation in animals receiving 3 boluses per week at 1.5 g/kg is higher than in those following other administration patterns. Additionally, the group receiving the highest dose of mannitol, 3 boluses per week (2.5 g/kg i.v.), exhibited the lowest Gd leakage values. The chronic effects of repetitive infusion on the BBB and brain metabolism remain unclear; however, studies on temporary BBB disruption have reported that excessive mannitol can cause widespread osmotic stress, leading to extensive neuronal damage beyond the intended BBB disruption. This non-specific damage complicates the attempts to isolate the effects of BBB opening and study specific mechanisms or outcomes, decreasing the reliability of experimental results and potentially altering the findings [15,16,17,18].

Of the metabolites assessed using MR spectroscopy, we observed that Gly and Cr levels in the striatum were higher in the group receiving 3 boluses per week at 1.5 g/kg compared to the group receiving 1 bolus per week at 2.0 g/kg. Conversely, Ala, Cho, and NAA levels decreased in the striatum after administering 1 bolus per week at 2.0 g/kg. Brain metabolites are often studied using techniques like MR spectroscopy to assess their levels in different neurological conditions where BBB dysfunction may be implicated [27,28,29]. Their levels can provide insights into the metabolic and functional status of the brain and its interactions with the BBB. However, the changes detected in the metabolites could also be involved the glymphatic system [30], which could not be determined in the present study. Ala plays a role in neurotransmitter metabolism and energy production in the brain. Its precise role in BBB function is not extensively studied, but alterations in its levels can reflect changes in the brain metabolism and neurotransmission [31]. Cho is crucial for the synthesis of acetylcholine, a neurotransmitter involved in various brain functions. Cho levels are indicative of membrane turnover and integrity, which are essential aspects of the BBB’s structure and function [32]. NAA is primarily found in neurons and is considered to be a marker of neuronal health and viability. Changes in NAA levels can indicate neuronal dysfunction or damage, which indirectly reflects on BBB integrity since a healthy BBB is crucial for neuronal homeostasis [33].

Unlike some current models of chronic BBB dysfunction that use high doses of neurotoxins causing extensive neuronal damage [6,7,8,9,10,11,12,13,14], our approach allows for the study of specific BBB-related changes without the added complexity of acute widespread neuronal injury. During this study, no brain lesions or anatomical differences were observed in association with the doses of mannitol used. On the other hand, the levels of all pro-inflammatory cytokines evaluated in the mannitol-induced rats were higher than at baseline, although no significant differences were observed. Pro-inflammatory cytokines such as TNF-α, IL-1β, IL-6, and IL-2 play several key roles in the brain, as follows: (i) They are crucial in initiating and regulating inflammation in response to infection, injury, or disease. (ii) These cytokines facilitate communication between the immune system and the nervous system, influencing brain function and behavior. (iii) They can affect the release and activity of neurotransmitters, impacting mood, cognition, and overall brain function. (iv) Pro-inflammatory cytokines can influence synaptic strength and plasticity, thereby affecting learning and memory processes. On the other hand, the expression of IL-10 in the brain serves several important functions: (i) IL-10 is a potent anti-inflammatory cytokine that helps suppress and regulate inflammatory responses in the brain, thereby protecting neural tissue from damage caused by excessive inflammation. (ii) It plays a crucial role in maintaining immune homeostasis by inhibiting the synthesis of pro-inflammatory cytokines and reducing the activation of microglia and astrocytes, the primary immune cells in the brain. (iii) IL-10 contributes to the repair processes in the brain by promoting the survival and regeneration of neurons and glial cells following injury or neurodegenerative processes [34,35,36].

A large number of studies have reported on the clinical and preclinical applications of the hyperosmotic agent mannitol. The systemic intravenous administration of mannitol is widely used clinically to reduce cerebral edema due to its osmotic properties [16,20,21]. It is typically administered intravenously in concentrations ranging from 5% to 25%, either as an intermittent bolus or continuous infusion, depending on the clinical situation. Dilution with other fluids allows for the adjustment of concentration based on specific patient needs. Close monitoring of the infusion rate is essential to prevent rapid fluid shifts, which can lead to complications such as pulmonary edema. When using higher concentrations (e.g., 20–25%), central venous catheterization is preferred to avoid irritation or damage to peripheral veins due to mannitol’s hypertonic nature.

Mannitol is a sugar alcohol that is not metabolized in the body, and, when administered intravenously, it increases the osmolarity of the blood. This osmotic effect draws water out of brain tissue cells, reducing swelling and pressure inside the skull. Thus, mannitol is effective in managing conditions such as traumatic brain injury, stroke, and certain neurological surgeries where cerebral edema can be a critical issue. In animal models of osmotic stress, such as with mannitol administration, there is an increase in cerebral blood volume and facilitation of tight junction opening in the BBB. Transient BBB opening is therefore a strategy used to facilitate the delivery of treatments into the brain for the effective management of CNS diseases.

Despite these studies, there have been no reported in vivo studies investigating the duration of osmotic BBB opening, or there is not a well-established chronic animal model specifically designed to induce BBB dysfunction using mannitol. Recently, time-lapse imaging has been employed to visualize hyperosmotic BBB opening in a tissue-engineered micro-vessel model using human stem-cell-derived brain microvascular endothelial cells [20]. The main findings of this study indicate that (i) the BBB opening is generally reversible within 2 h, and (ii) mannitol treatment induces stress in derived human brain microvascular endothelial cells, leading to disruptions in barrier function two days later, particularly with larger doses. These findings may elucidate the mechanisms underlying the chronic BBB dysfunction observed in our study.

Future investigations are necessary to (i) evaluate the mechanisms associated with this chronic BBB dysfunction, (ii) determine whether the increase in longitudinal assessment over 12 weeks is linked to progression in BBB dysfunction or the occurrence of brain lesions, (iii) assess the correlation between BBB dysfunction and potential cognitive impairment through behavioral testing, and (iv) correlate BBB dysfunction onset and progression with biomarkers.

Reproducible, simple, and accurate models of chronic BBB dysfunction, such as the one proposed in this work, are crucial for advancing our understanding of BBB dynamics in neurological disorders. They also hold promise for future therapeutic developments, including biomarkers and agents aimed at blocking BBB leakage.

## 4. Materials and Methods

### 4.1. Experimental Animals

All experimental animal procedures were conducted under procedure number: 15011/2021/003 approved by the Animal Care Committee, according to European Union Rules (Council Directive 2010/63/UE) and the Spanish regulation (RD 53/2013). Eighteen Sprague Dawley (SD) rats with a weight between 100 and 150 g were used for in vivo studies and randomly divided. Online software (Experimental Design Assistant; https://eda.nc3rs.org.uk/eda/login/auth, accessed on 20 January 2024) was used for the sample size calculation and the animal randomization. Animals were kept in a controlled environment at 22 ± 1 °C and 60 ± 5% humidity with 12:12 h light: darkness cycles and were fed ad libitum with standard diet pellets and tap water. As directed by the animal facility veterinarian, the rats were monitored daily for any signs of dehydration that may affect the animal’s wellbeing, including appearance, attitude, and response to a pinch test. All surgical procedures and MRI studies were conducted under sevoflurane (Abbott Laboratories, IL, USA) anesthesia (3–4%) using a carrier 65:35 gas mixture of N_2_O:O_2_.

### 4.2. Mannitol-Induced and Evaluation Chronic Disruption of Blood–Brain Barrier Integrity

To investigate whether successive intravenous (i.v.) tail injections of mannitol can create a model of chronic endothelial dysfunction, and considering temporary studies of BBB opening [15,16,17,18], the following experimental groups (*n* = 6 animals/group) with a study duration of 12 weeks were studied: (i) Group 1: 1 bolus/week (2.0 g/kg i.v., 25% solution); (ii) Group 2: 3 boluses/week (1.5 g/kg i.v., 25% solution); (iii) Group 3: 3 boluses/week (2.5 g/kg i.v., 25% solution). A longitudinal assessment of BBB leakage was conducted in the study groups using T1-weighted MRI every 3 weeks for 12 weeks. MRI studies were conducted at least 24 h after the respective mannitol injections. The potential association between i.v. mannitol and changes in brain metabolism related to chronic BBB dysfunction was examined using MR 1-H spectroscopy [27,28]. At the end of the study, potential brain lesions were assessed using a T2-weighted MRI in the final week of follow-up, which could appear as hypointense (dark) or hyperintense (bright) signals. The detection of lesions could indicate that mannitol administration protocols may cause brain damage, complicating the evaluation of BBB dysfunction. A histological confirmation of MRI-based BBB permeability findings was performed using Evans blue. Additionally, different neuroinflammation biomarkers were analyzed in the highest mannitol dose group (Group 3) every 3 weeks for 12 weeks (Figure 2).

### 4.3. Magnetic Resonance Imaging (MRI)

All studies were conducted on a Bruker Biospec 9.4 T MR scanner (horizontal bore magnet with 12 cm wide Bruker BioSpin, Bruker, Ettlingen, Germany) equipped with actively shielded gradients (440 mT m^−1^). Animals were imaged with a combination of a linear birdcage resonator (7 cm in diameter) for signal transmission and a 2 × 2 surface coil array for signal detection, positioned over the head of the animal, which was fixed with a teeth bar, earplugs, and adhesive tape. Transmission and reception coils were actively decoupled from each other.

MRI T1-maps: Rats were repeatedly scanned at five time points: before starting the series of injections (0), and at 3, 6, 9, and 12 weeks after the first mannitol injection. BBB permeability was determined using a step-down infusion protocol with a T1-shortening contrast agent (Gd, DOTAGRAF 0.5 mmol/mL, Bayer Hispania, Barcelona, Spain). Briefly, T1-weighted MR images (Rapid Acquisition with Relaxation Enhancement sequence (RARE)) with an echo time (ET) = 12.9 ms, rare factor (RF) = 3, 6 T1 experiments (480, 650, 940, 1500, 2400, and 9000 ms), 1 average, spectral bandwidth (SW) = 75 KHz, 18 consecutive slices of 0.7 mm, 16 × 16 mm^2^ field of view (FOV) with saturation bands to suppress signal outside this FOV, a matrix size of 128 × 128 (isotropic in-plane resolution of 125 μm/pixel × 125 μm/pixel), and implemented without a fat-suppression option. Images were acquired before and 45 min after the beginning of a 20 min step-down infusion with 0.2 M Gd (diluted in 0.9% NaCl; Gadovist^®^, Schering, Kenilworth, NJ, USA). Gd-injection was carried out via the tail vein, using a syringe pump (Model PhD2000, Harvard Apparatus, South Natick, MA, USA) programmed to reach the highest possible stable Gd blood concentration during 20 min. Contrast-induced signal changes were calculated from the scans taken before and after tracer infusion [12,23,24].

MRI T2-maps: The presence of brain lesions and the quantification of brain regions were determined from T2 maps, which were calculated from T2-weighted images acquired at 12 weeks using a multi-slice multi-echo (MSME) sequence: ET = 9 ms, RT = 3 s, 16 echoes with 9 ms echo spacing, flip angle = 180°, NA = 2, SW = 75 KHz, 14 slices of 1 mm, 19.2 × 19.2 mm^2^ FOV (with saturation bands to suppress signals outside this FOV), a matrix size of 192 × 192 (isotropic in-plane resolution of 100 µm/pixel × 100 µm/pixel), and implemented without the fat-suppression option.

MR spectroscopy: Three animals from each of Groups 1 and 2 were studied, with MR spectra obtained at baseline (before starting the injection series) and at 3, 6, 9, and 12 weeks. Spectroscopic analysis of different metabolites (alanine (Ala), creatine (Cr) with two peaks (at δ = 3.0 and 3.9 ppm, corresponding to blue and red hydrogens, respectively), g-aminobutyric acid (GABA), glutamate (Glu), glutamine (Gln), glycine (Gly), N-acetylaspartate (NAA), phosphocholine (PCh or Ch), and taurine (Tau)) in the cortex and striatum was performed as previously described [37]. Local shimming was performed by manual adjustment of first- and second-order shim coil currents using a proton-stimulated-echo acquisition mode (STEAM)-waterline sequence. The field homogeneity in a 3 × 3 × 3 mm^3^ voxel typically resulted in signal line widths of 10–20 Hz for the water signal. In vivo 1H magnetic resonance spectra of both hemispheres of the rat brain were acquired by using a STEAM-1H sequence with an echo time (ET) = 3 ms, mixing time (TM) = 10 ms, repetition time (RT) = 1500 ms, flip angle (FA) = 90°, number of averages (NA) = 128, cubic voxel 3 × 3 × 3 mm^3^ and acquisition time = 3:15 min. Water signal was suppressed by variable-power RF pulses with optimized relaxation delays.

### 4.4. MRI Data Analysis

Images were processed using ImageJ (Rasband WS, National Institutes of Health, Bethesda, MD, USA, http://rsb.info.nih.gov/ij/, accessed on 20 May 2024) on an independent computer workstation by a researcher blinded to the animal protocols.

For each animal, a Gd leakage map was created (Figure 2) by digitally subtracting the pre-contrast T1-weighted signal intensity from the post-contrast signal intensity and dividing by the pre-contrast signal intensity, as previously described [12,23,24]. Voxels above a threshold of 0.2 were manually delineated by a researcher blinded to the group assignments and projected onto the Gd leakage map, following the method described by van Vliet et al. [25,26]. For each ROI, the mean signal enhancement was calculated from the Gd-leakage map. The regions quantified included the whole brain, as well as the cortex, hippocampus, striatum, and corpus callosum separately according to a rat brain atlas.

At the end of the study at week 12, potential brain damage was assessed using T2 maps calculated from T2-weighted images, along with possible volume variations due to the different doses used.

Brain spectra of the cortex and striatum were processed using MestReNova software (MestReNova 11.0, Mestrelab Research, Santiago de Compostela, Spain). For the quantitative analysis, Ala, Cr, GABA, Glu, Gln, Gly, NAA, PCh, and Tau, were normalized to the lipid peak areas for each single spectrum [37].

### 4.5. Evans Blue

Study of the Evans blue extravasation was performed based on the protocol described by Goldim et al. [25,26]. Briefly, Evans blue (3 mL/kg; 2% *w*/*v*) was injected i.v. in tail vein under anesthesia. One hour later, rats were sacrificed and transcardially perfused with an ice-cold solution of PBS. The brain was dissected out, weighed, and stored at −80 °C until analysis.

Brain samples were put in the stove at 55 °C in N,N-dimethylformamide during 48 h to extract the Evans blue retained in the brain parenchyma after extravasation, and then centrifuged at 9000× *g* for 20 min. The absorbance of the supernatant was measured in plate reader at 630 nm.

### 4.6. Blood Samples and Analysis of Pro- and Anti-Inflammatory Cytokines

Blood samples were collected in test tubes (BD Microtainer SST Tubes. Ref: 365968, Franklin Lakes, NJ, USA) every 3 weeks before the MRI study, let them coagulate for 40 min and centrifuged at 1700× *g* (Ref. 5804, Eppendorf, Hamburg, Germany) for 7 min. The serum was removed and immediately frozen and stored at −80 °C.

The serum levels of pro-inflammatory cytokines (TNF-α, IL-1β, IL-6, and IL-2) and the anti-inflammatory cytokine (IL-10) in the high-dose group (3 bolus/week at 2.5 g/kg i.v., 25% solution), were measured using Milliplex MAP Rat Cytokine/Chemokine Magnetic Bead Panel kit according to the manufacturer’s instructions (Ref. RECYTMAG-65K, EMD Millipore, Darmstadt, Germany). Briefly, the assay utilizes 25 μL of sample to capture an analyte on color-coded magnetic beads coated with analyte-specific capture antibodies. Biotinylated detection antibodies are then added, followed by incubation with streptavidin–phycoerythrin. All measurements were conducted on a Bio-Plex 200 system (Bio-Rad), using Bio-plex manager 6.2 build 175 (Bio-Rad) software 3.0.

### 4.7. Statistical Analysis

All data were collected in a database created in Excel (Microsoft Office Excel 2016; Microsoft Corporation, Redmond, WA, USA) and were subsequently analyzed using SPSS software (IBM^®^ SPSS^®^ Statistics for Windows v20, SPSS Inc. Armonk, NY, USA). Data are reported as the mean and standard error of the mean (mean ± SEM). Statistical analysis of Gd-leakage maps data was performed using the Kruskal–Wallis test followed by a post hoc Mann–Whitney test. Spectroscopy and Evans blue dye data were analyzed using the Mann–Whitney test. Brain volume and body weight were analyzed using one-way ANOVA followed by Tukey’s honest significant test. Group differences were considered significant if *p* < 0.05 in all analyses. Regarding pro- and anti-inflammatory cytokines, basal levels represent the measurements taken at week 0, where each analyte value was normalized by dividing it by its corresponding baseline. Values from the subsequent weeks were also normalized to each animal’s baseline. Graphs were made using GraphPad Software (GraphPad Prism V.8.0.1, Boston, MA, USA).

## 5. Conclusions

Our data show chronic BBB dysfunction mainly in the cortex, hippocampus, and striatum, but not in the corpus callosum of rats under periodic mannitol dosing of 1 bolus/week (2.0 g/kg i.v., 25% solution) and 3 boluses/week (1.5 g/kg i.v., 25% solution) during 12 weeks. MR spectroscopy reported that significant relationships were observed between baseline levels and the mannitol dosing period in the striatum for the metabolites Ala, Cho, and NAA in the group receiving 1 bolus/week (2.0 g/kg i.v., 25% solution). Extravasation of Evans blue dye is consistent with MR results and indicates elevated values compared to healthy rats. No significant differences were found in the serum levels of all of the pro- and anti-inflammatory cytokines analyzed in the high-dose group receiving 3 boluses/week (2.5 g/kg i.v., 25% solution). Therefore, these results support the hypothesis that periodic i.v. administration of mannitol can induce chronic BBB dysfunction, making it a valuable tool for experimental or clinical studies aimed at elucidating the role of BBB in various disorders.

## Data Availability

The statistical analysis plan is available on request.

## Supplementary Materials

The following supporting information can be downloaded at: https://www.mdpi.com/article/10.3390/ijms25189792/s1.

## References

[1] Liebner S., Dijkhuizen R.M., Reiss Y., Plate K.H., Agalliu D., Constantin G. (2018). Functional morphology of blood-brain barrier in health and disease. Acta Neuropathol..

[2] Claude Luissint A., Artus C., Glacial F., Ganeshamoorthy K., Couraud P.O. (2012). Tight junctions at the blood brain barrier: Physiological architecture and disease-associated dysregulation. Fluids Barriers CNS.

[3] Zhen Z., Nelson A.R., Betsholtz C., Zlokovic B.V. (2015). Establishment and Dysfunction of the BBB. Cell.

[4] Curtaz C.J., Schmitt C., Herbert S.L., Feldheim J., Schlegel N., Gosselet F., Hagemann C., Roewer N., Meybohm P., Wöckel A. (2020). Serum-derived factors of breast cancer patients with brain metastases alter permeability of a human blood-brain barrier model. Fluids Barriers CNS.

[5] Appelt-Menzel A., Cubukova A., Günther K., Edenhofer F., Piontek J., Krause G., Stüber T., Walles H., Neuhaus W., Metzger M. (2017). Establishment of a Human Blood-Brain Barrier Co-culture Model Mimicking the Neurovascular Unit Using Induced Pluri- and Multipotent Stem Cells. Stem Cell Rep..

[6] Sanchez-Varo R., Mejias-Ortega M., Fernandez-Valenzuela J.J., Nuñez-Diaz C., Caceres-Palomo L., Vegas-Gomez L., Sanchez-Mejias E., Trujillo-Estrada L., Garcia-Leon J.A., Moreno-Gonzalez I. (2022). Transgenic Mouse Models of Alzheimer’s Disease: An Integrative Analysis. Int. J. Mol. Sci..

[7] Yokoyama M., Kobayashi H., Tatsumi L., Tomita T. (2022). Mouse Models of Alzheimer’s Disease. Front. Mol. Neurosci..

[8] Ghasemi A., Jeddi S. (2023). Streptozotocin as a tool for induction of rat models of diabetes: A practical guide. EXCLI J..

[9] Banks W.A., Gray A.M., Erickson M.A., Salameh T.S., Damodarasamy M., Sheibani N., Meabon J.S., Wing E.E., Morofuji Y., Cook D.G. (2015). Lipopolysaccharide-induced blood-brain barrier disruption: Roles of cyclooxygenase, oxidative stress, neuroinflammation, and elements of the neurovascular unit. J. Neuroinflammation.

[10] Yan B.C., Xu P., Gao M., Wang J., Jiang D., Zhu X., Won M.H., Su P.Q. (2018). Changes in the Blood-Brain Barrier Function Are Associated With Hippocampal Neuron Death in a Kainic Acid Mouse Model of Epilepsy. Front. Neurol..

[11] Procaccini C., Xu P., Gao M., Wang J., Jiang D., Zhu X., Won M.H., Su P.Q. (2015). Animal models of Multiple Sclerosis. Eur. J. Pharmacol..

[12] Van Vilet E.A., Ndode-Ekane X.E., Lehto L.J., Gorter J.A., Andrade P., Aronica E., Gröhn O., Pitkänen A. (2020). Long-lasting blood-brain barrier dysfunction and neuroinflammation after traumatic brain injury. Neurobiol. Dis..

[13] Alonso-Alonso M.L., Pérez-Mato M., Sampedro-Viana A., Correa-Paz C., Ávila-Gómez P., Sobrino T., Campos F., Castillo J., Iglesias-Rey R., Hervella P. (2022). Fibrin-Targeted Nanoparticles for Finding, Visualizing and Characterizing Blood Clots in Acute Ischemic Stroke. Pharmaceutics.

[14] Jones L.D., Jackson J.W., Maggirwar S.B. (2016). Modeling HIV-1 Induced Neuroinflammation in Mice: Role of Platelets in Mediating Blood-Brain Barrier Dysfunction. PLoS ONE.

[15] Xie J., Shen Z., Anraku Y., Kataoka K., Chen X. (2019). Nanomaterial-based blood-brain-barrier (BBB) crossing strategies. Biomaterials.

[16] Siegal T., Rubinstein R., Bokstein F., Schwartz A., Lossos A., Shalom E., Chisin R., Gomori J.M. (2000). In vivo assessment of the window of barrier opening after osmotic blood-brain barrier disruption in humans. J. Neurosurg..

[17] Joshi S., Ergin A., Wang M., Reif R., Zhang J., Bruce J.N., Bigio I.J. (2011). Inconsistent blood brain barrier disruption by intraarterial mannitol in rabbits: Implications for chemotherapy. J. Neurooncol..

[18] Van Vilet E.A., da Costa Araújo S., Redeker S., van Schaik R., Aronica E., Gorter J.A. (2007). Blood–brain barrier leakage may lead to progression of temporal lobe epilepsy. Brain.

[19] Rapoport S.I. (2000). Osmotic opening of the blood-brain barrier: Principles, mechanism, and therapeutic applications. Cell Mol. Neurobiol..

[20] Linville R.M., DeStefano J.G., Sklar M.B., Chu C., Walczak P., Searson P.C. (2020). Modeling hyperosmotic blood–brain barrier opening within human tissue-engineered in vitro brain microvessels. J. Cereb. Blood Flow. Metab..

[21] Kim J.H., Jeong H., Choo Y.H., Kim M., Ha E.J., Oh J., Shim Y., Kim S.B., Jung H.G., Park S.H. (2023). Optimizing Mannitol Use in Managing Increased Intracranial Pressure: A Comprehensive Review of Recent Research and Clinical Experiences. Korean J. Neurotrauma.

[22] Nishiyma A., Nishioka S., Islam S.M., Sakaguchi E. (2009). Mannitol lowers fat digestibility and body fat accumulation in both normal and cecectomized rats. J. Nutr. Sci. Vitaminol..

[23] Van Vilet E.A., Otte W.M., Gorter J.A., Dijkhuizen R.M., Wadman W.J. (2014). Longitudinal assessment of blood–brain barrier leakage during epileptogenesis in rats. A quantitative MRI study. Neurobiol. Dis..

[24] Van Vilet E.A. (2016). Blood–brain barrier leakage after status epilepticus in rapamycin-treated rats I: Magnetic resonance imaging. Epilepsia.

[25] Yen L.F., Wei V.C., Kuo E.Y., Lai T.W. (2013). Distinct Patterns of Cerebral Extravasation by Evans Blue and Sodium Fluorescein in Rats. PLoS ONE.

[26] Goldim M.P.S., Della Giustina A., Petronilho F. (2019). Using Evans Blue Dye to Determine Blood-Brain Barrier Integrity in Rodents. Curr. Protoc. Immunol..

[27] Gasparovic C., Prestopnik J., Thompson J., Taheri S., Huisa B., Schrader R., Adair J.C., Rosenberg G.A. (2013). 1H-MR spectroscopy metabolite levels correlate with executive function in vascular cognitive impairment. J. Neurol. Neurosurg. Psychiatry.

[28] Chaganti J., Zeng G., Tun N., Lockart I., Abdelshaheed C., Cysique L., Montagnese S., Brew B.J., Danta M. (2023). Novel magnetic resonance KTRANS measurement of blood-brain barrier permeability correlated with covert HE. Hepatol. Commun..

[29] Eva-Maria Ratai E.-M., González R.G. (2016). Clinical magnetic resonance spectroscopy of the central nervous system. Handb. Clin. Neurol..

[30] Jessen N.A., Munk A.S., Lundgaard I., Nedergaard M. (2015). The Glymphatic System—A Beginner’s Guide. Neurochem. Res..

[31] Dalangin R., Kim A., Campbell R.E. (2020). The Role of Amino Acids in Neurotransmission and Fluorescent Tools for Their Detection. Int. J. Mol. Sci..

[32] Derbyshire E., Obeid R. (2020). Choline, Neurological Development and Brain Function: A Systematic Review Focusing on the First 1000 Days. Nutrients.

[33] Schuff N., Meyerhoff D.J., Mueller S., Chao L., Sacrey D.T., Laxer K., Weiner M.W. (2006). N-acetylaspartate as a marker of neuronal injury in neurodegenerative disease. Adv. Exp. Med. Biol..

[34] Zhang W., Xiao D., Mao Q., Xia H. (2023). Role of neuroinflammation in neurodegeneration development. Signal Transduct. Target. Ther..

[35] Al-Qahtani A.A., Alhamlan F.S., Al-Qahtani A.A. (2024). Pro-Inflammatory and Anti-Inflammatory Interleukins in Infectious Diseases: A Comprehensive Review. Trop. Med. Infect. Dis..

[36] McCabe J.J., Walsh C., Gorey S., Harris K., Hervella P., Iglesias-Rey R., Jern C., Li L., Miyamoto N., Montaner J. (2024). C-Reactive Protein, Interleukin-6, and Vascular Recurrence According to Stroke Subtype: An Individual Participant Data Meta-Analysis. Neurology.

[37] Dopico-López A., Pérez-Mato M., da Silva-Candal A., Iglesias-Rey R., Rabinkov A., Bugallo-Casal A., Sobrino T., Mirelman D., Castillo J., Campos F. (2021). Inhibition of endogenous blood glutamate oxaloacetate transaminase enhances the ischemic damage. Transl. Res..

