# Supplementary material for: Assessment of Mannitol-Induced Chronic Blood–Brain Barrier Dysfunction In Vivo Using Magnetic Resonance
Source: Int J Mol Sci. 2024 Sep 10;25(18):9792. doi: 10.3390/ijms25189792 (PMC11431755; doi:10.3390/ijms25189792)
Supplement: Supplementary file 1 [file ijms-25-09792-s001.zip › ijms-3164648-supplementary.pdf]

International Journal of
Molecular Sciences

MDPI

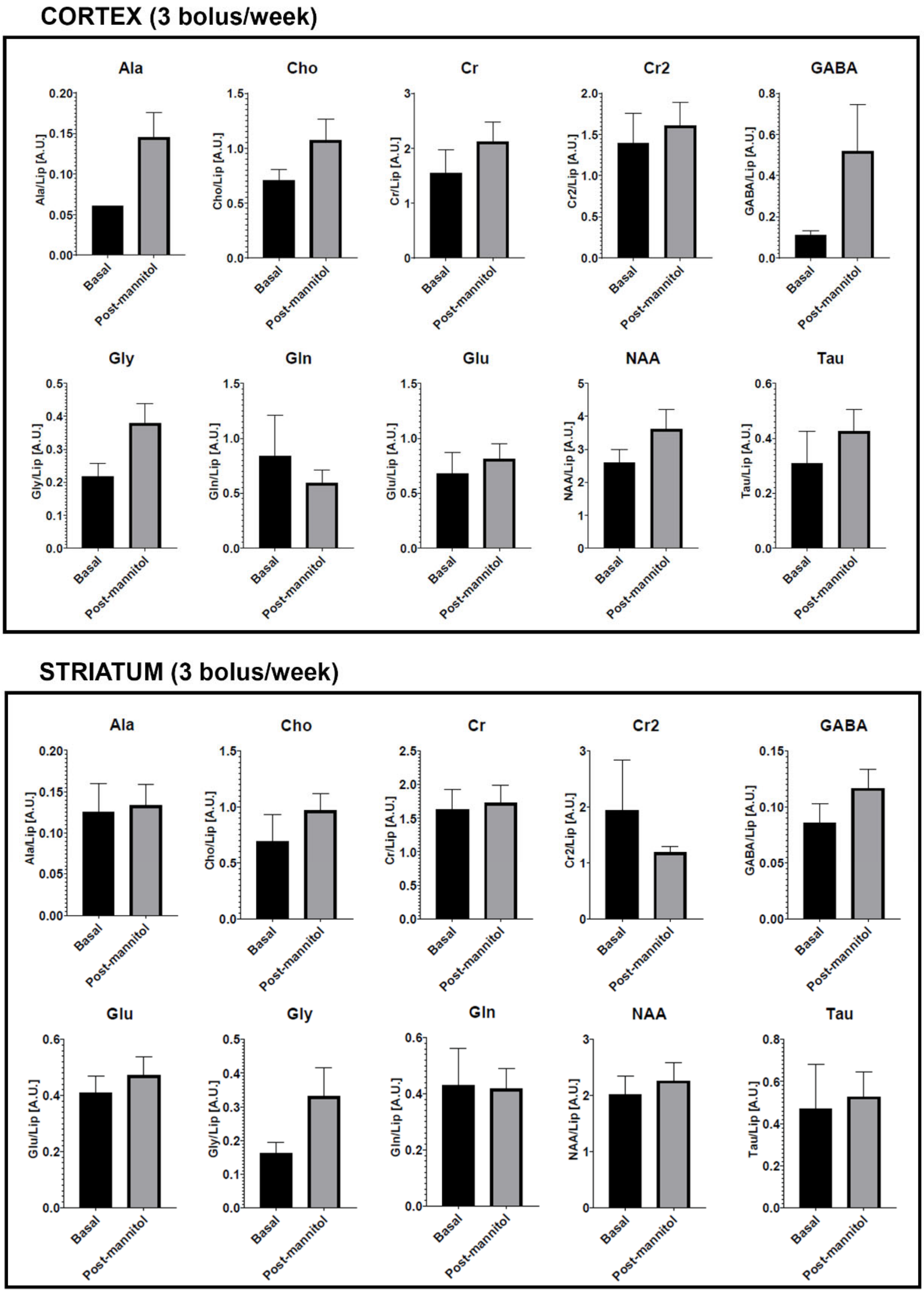

Supplemental Figure S1. Metabolite differences in group 2 (3 bolus/week (1.5 g/kg i.v., 25% solution)), *p<0.05. Metabolites: alanine (Ala), creatine (Cr), g-aminobutyric acid (GABA), glutamate (Glu), glutamine (Gln), glycine (Gly), N-acetylaspartate (NAA), phosphocholine (PCh or Ch), and taurine (Tau).
